# Impaired Cytotoxic Response in PBMCs From Patients With COVID-19 Admitted to the ICU: Biomarkers to Predict Disease Severity

**DOI:** 10.3389/fimmu.2021.665329

**Published:** 2021-05-26

**Authors:** Lorena Vigón, Daniel Fuertes, Javier García-Pérez, Montserrat Torres, Sara Rodríguez-Mora, Elena Mateos, Magdalena Corona, Adolfo J. Saez-Marín, Rosa Malo, Cristina Navarro, María Aranzazu Murciano-Antón, Miguel Cervero, José Alcamí, Valentín García-Gutiérrez, Vicente Planelles, María Rosa López-Huertas, Mayte Coiras

**Affiliations:** ^1^ Immunopathology Unit, National Center of Microbiology, Instituto de Salud Carlos III, Madrid, Spain; ^2^ School of Telecommunications Engineering, Universidad Politécnica de Madrid, Madrid, Spain; ^3^ Hematology Service, Hospital Universitario Ramón y Cajal, Madrid, Spain; ^4^ Neumology Service, Hospital Universitario Puerta de Hierro, Majadahonda, Spain; ^5^ Neumology Service, Hospital de El Escorial, El Escorial, Spain; ^6^ Family Medicine, Centro de Salud Doctor Pedro Laín Entralgo, Alcorcón, Spain; ^7^ Internal Medicine Service, Hospital Universitario Severo Ochoa, Leganés, Spain; ^8^ Division of Microbiology and Immunology, University of Utah School of Medicine, Salt Lake City, UT, United States

**Keywords:** SARS-CoV-2, COVID-19, cytotoxic response, NK and NKT cells, CD8 lymphocytes +

## Abstract

Infection by novel coronavirus SARS-CoV-2 causes different presentations of COVID-19 and some patients may progress to a critical, fatal form of the disease that requires their admission to ICU and invasive mechanical ventilation. In order to predict in advance which patients could be more susceptible to develop a critical form of COVID-19, it is essential to define the most adequate biomarkers. In this study, we analyzed several parameters related to the cellular immune response in blood samples from 109 patients with different presentations of COVID-19 who were recruited in Hospitals and Primary Healthcare Centers in Madrid, Spain, during the first pandemic peak between April and June 2020. Hospitalized patients with the most severe forms of COVID-19 showed a potent inflammatory response that was not translated into an efficient immune response. Despite the high levels of effector cytotoxic cell populations such as NK, NKT and CD8+ T cells, they displayed immune exhaustion markers and poor cytotoxic functionality against target cells infected with pseudotyped SARS-CoV-2 or cells lacking MHC class I molecules. Moreover, patients with critical COVID-19 showed low levels of the highly cytotoxic TCRγδ+ CD8+ T cell subpopulation. Conversely, CD4 count was greatly reduced in association to high levels of Tregs, low plasma IL-2 and impaired Th1 differentiation. The relative importance of these immunological parameters to predict COVID-19 severity was analyzed by Random Forest algorithm and we concluded that the most important features were related to an efficient cytotoxic response. Therefore, efforts to fight against SARS-CoV-2 infection should be focused not only to decrease the disproportionate inflammatory response, but also to elicit an efficient cytotoxic response against the infected cells and to reduce viral replication.

## Introduction

The novel coronavirus named as severe acute respiratory syndrome coronavirus 2 (SARS-CoV-2) emerged in late 2019 and it is responsible for one of the biggest pandemics in the recent History (1). SARS-CoV-2 is a single-stranded RNA virus that causes coronavirus disease 2019 (COVID-19). It was firstly described in December 2019, when several cases of an atypical pneumonia with unknown origin were described in Wuhan (Hubei Province, China) (2). This is the third coronavirus with pandemic potential that spreads through the world population since 2002, when a contagious infectious respiratory disease was reported in Guangdong (China) (3). This first SARS pandemic was caused by a new coronavirus termed SARS-CoV that showed no genetic relationship with any known human coronaviruses (4, 5). And it was followed in 2012 by the onset of the Middle East Respiratory Syndrome (MERS) in Saudi Arabia that was caused by the new coronavirus MERS-CoV (6). SARS-CoV-2 has been shown to be superior to other coronaviruses in its rapid growth and high capacity for dissemination within the population (7).

Clinical presentations of COVID-19 are very different and vary from mild to critical disease. Mild or self-limited COVID-19 has been described when there is no pneumonia or it has a mild course. These patients generally survive to the infection with no need of hospitalization. In the case of severe or progressive disease, the infected patients develop pulmonary damage characterized by bilateral infiltrates, as well as dyspnea and hypoxia that require hospitalization and supplemental oxygen. Finally, some patients develop critical or fatal COVID-19 that also includes respiratory failure from acute respiratory distress syndrome (ARDS) (8), as well as shock and/or hypercytokinaemia with multiorgan dysfunction (2). Therefore, in the most severe presentations of COVID-19, patients develop a cytokine storm syndrome similar to the haemophagocytic lymphohistiocytosis (sHLH) disease, which reveals a state of hyperinflammation triggered by the viral infection (9, 10). All these events lead to invasive mechanical ventilation and eventually, to the patient’s decease (11, 12). Fortunately, not all people infected with SARS-CoV-2 develop severe respiratory illness as most infections remain essentially mild (2, 13, 14). The reasons for the evolution of these different clinical presentations are still largely unknown. Since an effective treatment and vaccine are not yet available for everyone, there is an imminent need to better understand COVID-19, not only to control the pandemic spread, but also to identify accurate biomarkers that may help to identify as soon as possible those patients at-risk to develop the most severe, fatal form of COVID-19 in order to take the most adequate preventive measures. To this aim, the characterization of the immune response developed by each patient against SARS-CoV-2 infection is essential. It has been described an impairment in the cellular immune response during SARS-CoV-2 infection, with low functionality of macrophages and CD4 cytopenia, defective NK cell functions and T-cell exhaustion, together with inappropriate type I IFN responses and massive inflammatory cytokine production (15, 16). Surprisingly, most patients, including those with critical COVID-19, develop measurable neutralizing antibodies after infection, suggesting that the humoral adaptive immunity is still functional (17). All these data suggest that SARS-CoV-2 may partially evade the immune response in some patients and that this could be related to the severity of the disease (16). In fact, cellular adaptive immune response has been described as critical for an optimal outcome after the infection (18).

Spain was one of the European countries more severely affected by the first wave of COVID-19 pandemic, with approximately 100 excess deaths per 100,000 people, equivalent to 38% relative increase of people who would not have died if the pandemic had not occurred (19). A case fatality rate of 9.6% was registered in Spain by the end of the first pandemic peak on March 2020, which was higher than the average rate observed in other neighboring countries (7.3 ± 5.1%) (updated on August 3rd 2020 by the Spanish Ministry of Health) (20). This rate was nearly 3 times higher than that observed in other countries with much more cases such as the USA, with 15 times more confirmed cases than Spain but with a fatality rate of 3.3% on the same date. It is still unclear why there was a higher lethality by SARS-CoV-2 in Spain in comparison with other European countries with similar population density. However, despite the dramatic and early progression of the pandemic in Spain, seroprevalence appeared to be quite low by the end of the first pandemic peak, as it was estimated to be 5% on average at the time, although it was more than five times higher in hotspot areas such as Madrid (21). Therefore, in this study we characterized the immune cellular response against SARS-CoV-2 developed by Spanish patients with different presentations of COVID-19 who were recruited in hotspot locations by the end of the first pandemic peak in order to understand the disproportionate effect of SARS-CoV-2 in this country, as well as to determine the best biomarkers to predict the severity of COVID-19 evolution.

## Methods

### Study Subjects

Blood samples were obtained from 109 patients with COVID-19 who developed different presentations of the disease (**Table 1**). All patients were recruited in Madrid (Spain) between April and June 2020, right after the first pandemic peak (estimated at the end of March). The inclusion criterion that all individuals had to meet was that they had a positive SARS-CoV-2 RT-qPCR assay in naropharyngeal smear, which was performed in every hospital according to internal validated protocols. The exclusion criterion was that individuals younger than 18 years old could not participate in the study. Fifty-five patients showed mild COVID-19 and required Primary Healthcare attention and home isolation until the PCR assay for SARS-CoV-2 was negative. Fifty-four patients required hospitalization due to lung complications and were further categorized as either severe (n=19) or critical (n=35) COVID-19. Admission to the Intensive Care Unit (ICU) was necessary for patients with critical disease, although two patients who deceased before being admitted to ICU were also considered critical. Healthy donors (n=20) with similar age and gender distribution as the patients with COVID-19 were recruited as basal controls. The inclusion criteria of healthy donors were that they had not been infected with SARS-CoV-2 and that they matched age and gender of patients with COVID-19. Timing of samples collection was affected by the confinement decreed by the Spanish Government, mostly due to the recruitment of patients with mild COVID-19 who were homebound was delayed until the end of the confinement.

**Table 1:** Summary of clinical data of patients with COVID-19 that participated in the study.

	Mild COVID-19	Severe COVID-19	Critical COVID-19
Patients (n)	55	19	35
Median age with IQR (years)	46 (29.3 to 56.3)	72 (67.0 to 84.0)	63 (56.0 to 71.0)
Male/female (n)	18/37	12/7	26/9
Hypertension (Yes/No/Und)	7/48/0	13/6/0	20/13/2
Dyslipemia (Yes/No/Und)	10/45/0	6/13/0	12/21/2
Diabetes (Yes/No/Und)	1/54/0	5/14/0	5/28/2
Pneumonia (Yes/No/Und)	0/55/0	15/4/0	28/3/4
Invasive mechanical ventilation (Yes/No/Und)	NA	1/18/0	27/6/2
DIC (Yes/No/Und)	0/55/0	3/14/0	4/27/2
Median LOS with IQR (days)	NA	23 (10.0 to 38.0)	50 (34.0 to 92.3)
Median ICU stay with IQR (days)	NA	0	48 (20.5 to 62.0)
Exitus (Yes/No)	0/55	0/19	11/24

DIC, Disseminated intravascular coagulation; LOS, Length of hospitalization; ICU, Intensive Care Unit; IQR, Interquartile range; LOS, Length of hospitalization stay; NA, Not applicable; Und, Undetermined.

All clinical data from non-hospitalized and hospitalized patients are summarized in **Supplemental Table 1** and **Supplementary Table 2**, respectively.

### Cells

Peripheral blood mononuclear cells (PBMCs) were isolated from blood samples by centrifugation through Ficoll-Hypaque gradient (Pharmacia Corporation, North Peapack, NJ). Plasma and cells were cryopreserved until the moment of analysis. Due to the limited number of cells isolated per sample, not all the analyses were performed with all the samples.

K562 cell line (ECACC 89121407) was kindly provided by Dr. Cristina Eguizabal (Basque Centre Transfusions and Human Tissue, Álava, Spain) and these cells were cultured in RPMI 1640 medium supplemented with 10% (v/v) fetal calf serum (FCS), 2mM L-glutamine, 100µg/ml streptomycin, 100UI/ml penicillin (Lonza, Basel, Switzerland). Vero E6 (African green monkey kidney) cell line (ECACC 85020206) was kindly provided by Dr. Antonio Alcami (CBM Severo Ochoa, Madrid). Vero E6 and HEK-293T (National Institute for Biological Standards and Control [NIBSC]) cells were cultured in DMEM supplemented with 10% FCS, 2 mM L-glutamine and 100 units/ml penicillin and streptomycin (Lonza).

### Antibodies and Flow Cytometry

Conjugated antibodies for surface staining CD3-APC, CD4-PercP, CD8-APC-H7, CD16-PercP, CD56-FITC, CD107a-PE-Cy7, NKp44-BUV395, and NKp46-BUV650 were purchased from BD Biosciences (San Jose, CA), whereas PD1-BUV650, NKG2A-PE, and NKG2C-Alexa Fluor700 were purchased from R&D Systems (Minneapolis, MN). Tregs cells were characterized by staining with CD4-PercP, CD25-PE-Cy5 and CD127-FITC (R&D Systems). CD4+ and CD8+ T cell subpopulations were determined by staining with CCR7-FITC and CD45RA-PE-Cy7 (Biosciences) as follows: naïve (CD45RA+CCR7+), central memory (TCM) (CD45RA-CCR7+), effector memory (TEM) (CD45RA-CCR7-) and terminally differentiated effector memory (TEMRA) (CD45RA+CCR7-) cells.

For intracellular staining of granzyme B (GZB) from NK and NKT cells, PBMCs were treated for 4 h at 37°C with Hsp70 peptide 1 µgr/ml (Abcam, Cambridge, UK) to stimulate cytolytic activity of NK cells, in the presence of brefeldin A (BD Biosciences). Cells were then stained with antibodies against CD3, CD56 and CD16 conjugated to APC, FITC and PercP, respectively. After fixation and permeabilization with IntraPrep Permeabilization Reagent (Beckman Coulter), cells were stained with an antibody against GZB-PE (BD Biosciences) and then acquired and analyzed in a BD LSRFortessa X-20 flow cytometer (BD Biosciences) using FACS Diva (BD Biosciences) and FlowJo_V10 software (TreeStar).

### Luminex Assay

A customized Human Magnetic Luminex Assay kit (R&D Systems) was used for the simultaneous detection of the following cytokines in plasma: IL1β, IL2, IL2R, IL6, IL7, IL8, IL10, IL12, IL17, IL21, CD14, TNFα, MIP-1α, MIP-1β, GM-CSF, IFNα, IFNβ, IFNγ. Instructions provided by the supplier were followed and analysis was performed on a Bio-Plex 200 System (Bio-Rad).

### Pseudotyped SARS-CoV-2 Infection Assay

Infections with SARS-CoV-2 were performed using a one-cycle pseudotyped virus encoding the reporter gene *renilla* and SARS-CoV-2 S glycoprotein within an HIV-1 ∆*env* genome (pNL4-3Δ*env*Ren), which was generated as previously described (22). The codon-optimized cDNA encoding G614 SARS-CoV-2 S glycoprotein (QHU36824.1) without the last 19 amino acids (23) was synthesized by GeneArt Gene Synthesis (ThermoFisher Scientific España, Madrid, Spain), amplified by PCR and cloned into pcDNA3.1 expression vector. Due to D614 viruses were the majority of the earliest variants detected in Spain within clade 19B (24), a mutant clone introducing D614G change was created by site-directed mutagenesis and DNA sequence was confirmed. Vero E6 were infected with equal amounts of both pseudoviruses (D614 and G614) (100 ng p24 Gag/well) and then plated onto 48-well plates. After 48 hours of incubation, Vero cells were washed and co-cultured 1:10 for 1 hour with PBMCs from patients with different presentations of COVID-19. Vero monolayer was then detached with trypsin-EDTA solution (Sigma Aldrich-Merck, Darmstadt, Germany) and caspase-3 activity was measured by luminescence using Caspase-Glo 3/7 Assay system (Promega).

### NK Cell Cytotoxicity Assay

K562 cells were stained with PKH26 Red Fluorescence Cell Linker kit (Sigma Aldrich-Merck) and used as target cells for NK cells in PBMCs from patients with COVID-19. Stained K562 cells were co-cultured with PBMCs from these patients (1:10) for 1 hour. Cells were then collected and Annexin V conjugated with FITC (Thermofisher) was used to measure early apoptosis by flow cytometry.

### HLA-E Genotyping

Whole genomic DNA was isolated from PBMCs using QIAamp DNA Blood Mini Kit (Qiagen Iberia, Madrid, Spain). HLA-E genotyping was performed by StepOne Real-Time PCR System (Thermo Fisher Scientific, Waltham, MA) using 100 ng of genomic DNA to differentiate between alleles HLA-E*0101 and *0103. In-house primers and PrimeTime locked nucleic acids (LNA) probes were supplied by IDT (Integrated DNA Technologies, Leuven, Belgium): Primer forward: 5’-GCAGTGGATGCATGGCT-3’; Primer reverse: 5’-GGTCCTCATTCAGGGTGAGATA-3’; Probe_A-FAM (HLA-E*0101 allele): 5’-CGC+C+T+GTC+GG-3’; Probe_G-YAK (HLA-E*0103 allele): 5’-CGC+C+C+GTCGG-3’.

### KIR Haplotyping

Genomic DNA was extracted as described above. Killer cell Ig-like receptors (KIR) genotyping was performed with 360 ng of genomic DNA using LinkSeq KIR Typing kit (One Lambda, Thermo Fisher Scientific), which allows testing all 15 human KIR genes and 2 pseudogenes, including both full length and deleted forms of 3DP1 and 2DS4 and allele-specific variants in 3DL1, 2DL1. qPCR was performed using StepOne Real-Time PCR System (Thermofisher Scientific) according to manufacturer’s instructions. These data were exported to SureTyper software (One Lambda) for interpretation and identification of KIR genotypes. KIR haplotypes AA and BX were assigned to each patient sample as previously shown (25). B haplotypes are defined as those carrying one or more of the following genes: KIR2DL2, -2DL5, -3DS1, -2DS1, -2DS2, -2DS3 and -2DS5; whereas A haplotypes are defined as those that do not carry any of these genes.

### Random Forest Algorithm

A Random Forest algorithm (26) was applied to predict the categorization of patients with different presentations of COVID-19 into mild, severe and critical disease and to evaluate the resulting accuracy. The selection of these parameters was performed according to the existence of significant statistical differences between groups (p<0.05) as follows: regarding plasma levels of cytokines: IL-6, TNF-α, IL-2, CD25/IL2-Rα, IFNα, and IL12-p70; regarding NK and NKT cells: total CD56+, CD3+CD56+CD16+GZB+, and unspecific direct cytotoxicity against target cells without expression of MHC Class I molecules (missing-self) (annexin V); genetic parameters: HLAE 101/101, 103/103 and 101/103, haplotypes KIR AA and BX; regarding T cells: total CD4+ T cells, Tregs, CD8 ± TCRγδ+, and cytotoxicity against SARS-CoV-2 infected cells (caspase-3 activity on the target cells). Data from 79 patients with different presentations of COVID-19 were included in the analysis. In order to avoid bias in the selection of training, testing and validation sets, we performed a combined feature selection and classification procedure using a Random Forest classifier with a nested 5-fold cross validation procedure for each competing algorithm, as previously described (27, 28). The relative importance for each feature in the categorization of patients with COVID-19 was calculated by Gini VIM method (29).

### Statistical Analysis

Statistical analysis was performed using Graph Pad Prism 8.0 (Graph Pad Software Inc., San Diego, CA). Statistical significance was calculated using one-way ANOVA and Tukey’s multiple comparisons test. P values (*p*) < 0.05 were considered statistically significant in all comparisons and were represented as *, **, ***, or **** for *p*<0.05, *p*<0.01, *p*<0.001, or *p*<0.0001, respectively.

## Results

### Patients’ Cohorts

Observational, cross-sectional study that included 109 patients diagnosed with COVID-19. Most patients (67%) who developed mild symptoms (henceforth, Mild COVID-19) were females, whereas 70% of patients with severe (henceforth, Severe COVID-19) or critical (henceforth, Critical COVID-19) COVID-19 were males. The median age of patients with mild COVID-19 was 46 years (interquartile range (IQR) 29.3 to 56.3 years), whereas the median age of patients with severe or critical COVID-19 was 66.5 years (IQR 58.5 to 73.0 years). Twenty healthy donors (40% male, 60% female) with median age 55.5 years (IQR 48.5 to 60.8 years) were recruited as basal controls. Sixty percent of patients with severe or critical COVID-19 had previous hypertension, 31% showed dyslipemia and 15% had diabetes, in comparison with patients with mild disease that had 13% hypertension, 18% dyslipemia, and 2% diabetes. All patients received the appropriate treatment for these underlying diseases. Eighty percent of hospitalized patients had pneumonia and 52% required invasive mechanical ventilation. Thirteen percent of severe and critical patients developed disseminated intravascular coagulation (DIC), but this coagulopathy was not observed in patients with mild COVID-19. Median length of hospitalization stay was 23 days (IQR 10.0 to 38.3 days) for patients with severe COVID-19, whereas patients with critical COVID-19 stayed at the hospital for a median of 50 days (IQR 34.0 to 92.3 days). Patients with severe COVID-19 were not admitted to ICU; the median ICU stay for patients with critical COVID-19 was 48 days (IQR 20.5 to 62.0 days). Thirty-one percent of patients with severe COVID-19 deceased during hospitalization, whereas all patients with mild and severe COVID-19 recovered from the infection. Due to the confinement decreed by the Spanish Government between March 14^th^ and June 21^st^ 2020, hospitalized patients with severe and critical COVID-19 were recruited in April and May, whereas patients with mild COVID-19 who were homebound, had to be recruited by the end of June, once the confinement ended. Therefore, the median number of days from clinical onset to sampling was 43.5 days for critical COVID-19 (IQR 27.3 to 53.0), 26.5 days for severe COVID-19 (IQR 13.0 to 37.8) and 87.5 days for mild COVID-19 (IQR 81.3 to 95.0).

Treatment for patients with mild COVID-19 was symptomatic. Patients with severe and critical COVID-19 were treated with hydroxychloroquine (94.7 and 88.6%, respectively), lopinavir/ritonavir (42.1 and 71.4%, respectively), azithromycin (78.9 and 80%, respectively), corticosteroids (68.4 and 88.6%, respectively), tocilizumab (36.8 and 62.9%, respectively), and low-molecular-weight heparin (LMWH) (31.6 and 71.4%, respectively).

### Cytokine Profile in Plasma of Patients With Different Stages of COVID-19

**Figure 1:**
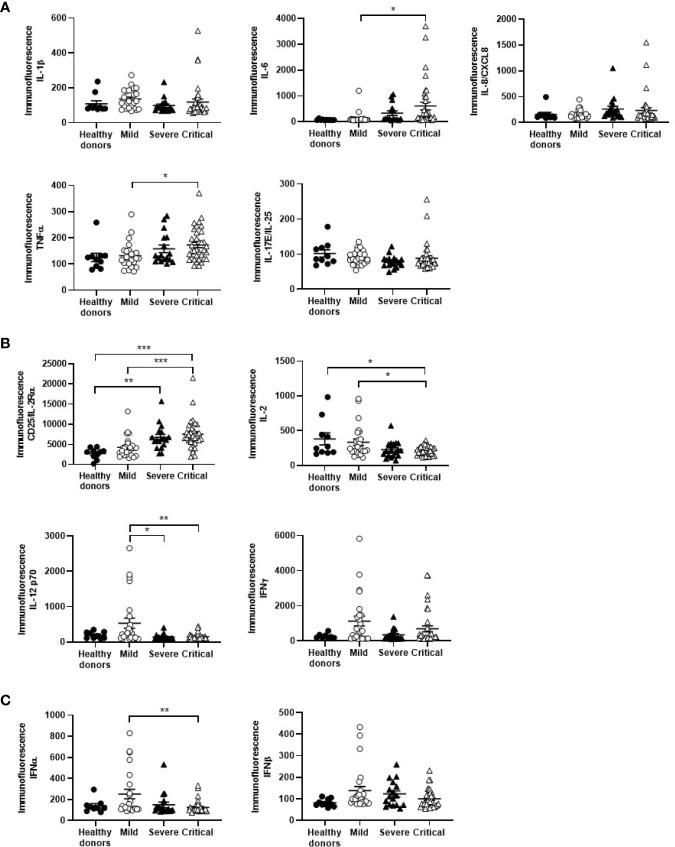
Cytokine profile in plasma of patients with different stages of COVID-19. The levels of chemokines and proinflammatory cytokines **(A)**, cytokines involved in Th1 differentiation and survival **(B)**, and cytokines with antiviral activity **(C)** were analyzed in plasma of patients with mild, severe and critical COVID-19, in comparison with healthy donors. Each dot corresponds to one sample and lines represent mean ± standard error of the mean (SEM). Statistical significance was calculated using one-way ANOVA and Tukey’s multiple comparisons test. *p < 0.05; **p < 0.01; ***p < 0.005.

The role of cytokines in the severity of COVID-19 presentations has been described before (30, 31). Chemokines such as IL-8/CXCL8 and proinflammatory cytokines such as IL-6 (p<0.05) and TNFα (p<0.05) were increased 1.4-, 4.7-, and 1.3-fold, respectively, in the plasma of patients with critical COVID-19, in comparison with patients with mild disease (**Figure 1A**). Soluble CD25/IL-2Rα was also increased 1.8-fold in patients with severe and critical (p<0.001) COVID-19, regarding mild COVID-19, whereas the amount of IL-2 in plasma was reduced 1.5-fold (p<0.05 for critical COVID-19) (**Figure 1B**). Other cytokines also involved in Th1 differentiation and survival such as IL-12 were reduced 4.0- and 3.7-fold in patients with severe (p<0.05) and critical (p<0.01) COVID-19, respectively, as well as IFNγ, which was also reduced 3.4- and 1.6-fold, respectively. Other IFNs with antiviral activity such as type I IFNα (p<0.01) and β were reduced 2.0- and 1.4-fold, respectively, in critical COVID-19 in comparison with mild disease (**Figure 1C**).

### CD4+ T Cell Subpopulations During Severe and Critical COVID-19

**Figure 2:**
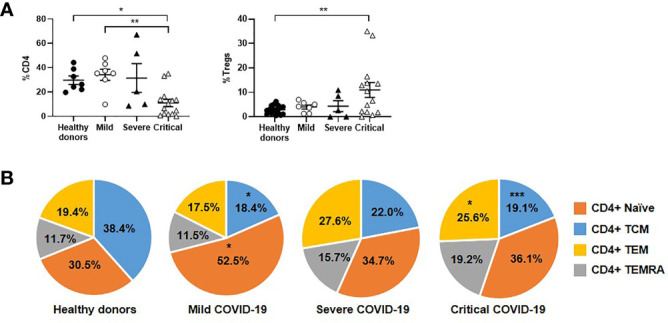
Analysis of CD4+ T cell subpopulations in patients with different stages of COVID-19. The levels of CD4+ T cells and Tregs **(A)**, as well as the distribution of CD4 subpopulations **(B)**, were analyzed in PBMCs of patients with mild, severe and critical COVID-19, in comparison with healthy donors. Each dot corresponds to one sample and lines represent mean ± SEM. Statistical significance was calculated using one-way ANOVA and Tukey’s multiple comparisons test. *p < 0.05; **p < 0.01.

Patients with critical COVID-19 had levels of peripheral CD4+ T cells that were reduced 3.1-fold in comparison with mild COVID-19 (p<0.01) and healthy donors (p<0.05), whereas the levels of Tregs were increased 2.7-fold in these patients (p<0.05 regarding healthy donors) (**Figure 2A**). The levels of effector CD4+ T cell subpopulations TEM and TEMRA were increased 1.5-fold in patients with severe and critical COVID-19, in comparison with mild COVID-19 (**Figure 2B**). Naïve CD4+ T cell population was reduced 1.5-fold in patients with severe and critical disease, in comparison with mild COVID-19.

### Changes in CD8+ T Cell Subpopulations and Cytotoxic Activity Against SARS-CoV-2-Infected Cells

**Figure 3:**
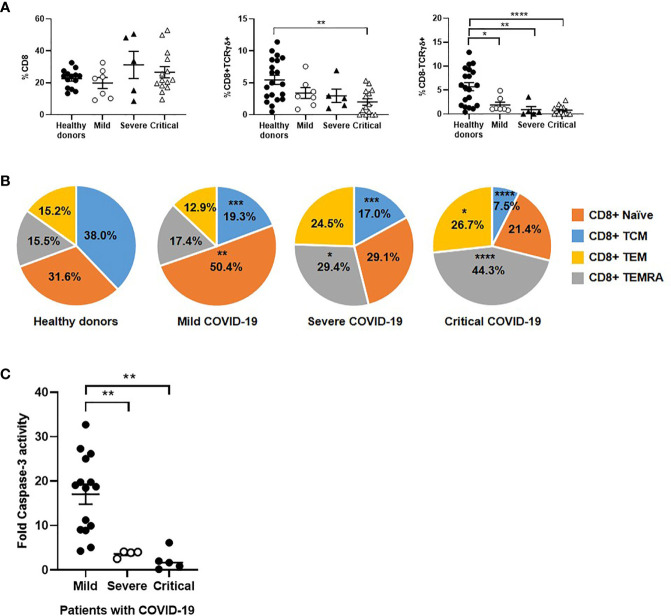
Analysis of CD8+ T cell subpopulations in patients with different stages of COVID-19. The levels of classical CD8+ T cells and unconventional CD8 ± TCRγδ+ **(A)**, as well as the distribution of CD8 subpopulations **(B)**, were analyzed in PBMCs of patients with mild, severe and critical COVID-19, in comparison with healthy donors. **(C)** Antiviral cytotoxicity of PBMCs from patients with different presentations of COVID-19 was analyzed by quantifying caspase-3 activity in a monolayer of Vero E6 cells infected with pseudotyped SARS-CoV-2 viruses D614 and G614 that were co-cultured with PBMCs (1:10) for 1 hour. Each dot corresponds to one sample and lines represent mean ± SEM. Statistical significance was calculated using one-way ANOVA and Tukey’s multiple comparisons test. *p < 0.05; **p < 0.01; ***p < 0.005; ****p < 0.001.

Unlike CD4+ T cells, the number of CD8+ T cells was increased 1.6- and 1.3-fold in severe and critical COVID-19, respectively, in comparison with patients with mild disease (**Figure 3A**). However, the levels of CD8+ T cells expressing TCRγδ were reduced 1.7-fold in patients with critical COVID-19, whereas CD8-TCRγδ+ cells were reduced 2.4-fold in these patients, in comparison with mild COVID-19. Subpopulations of effector CD8+ T cells TEM and TEMRA were increased 2.1- and 2.4-fold in patients with severe and critical COVID-19, respectively, in comparison with patients with mild disease (**Figure 3B**). The expression levels of TEM and TEMRA CD8+ T cells in mild patients were similar to controls.

The specific activity of effector cytotoxic cells against SARS-CoV-2 was tested in a monolayer of Vero E6 cells infected with pseudotyped SARS-CoV-2 viruses D614 and G614, and it was reduced 4.7- (p<0.01) and 7.8- (p<0.01) fold in patients with severe and critical COVID-19, respectively, in comparison mild COVID-19 (**Figure 3C**).

### NK and NKT Cell Population Distribution and Direct Cytotoxic Activity Against Target Cells

**Figure 4:**
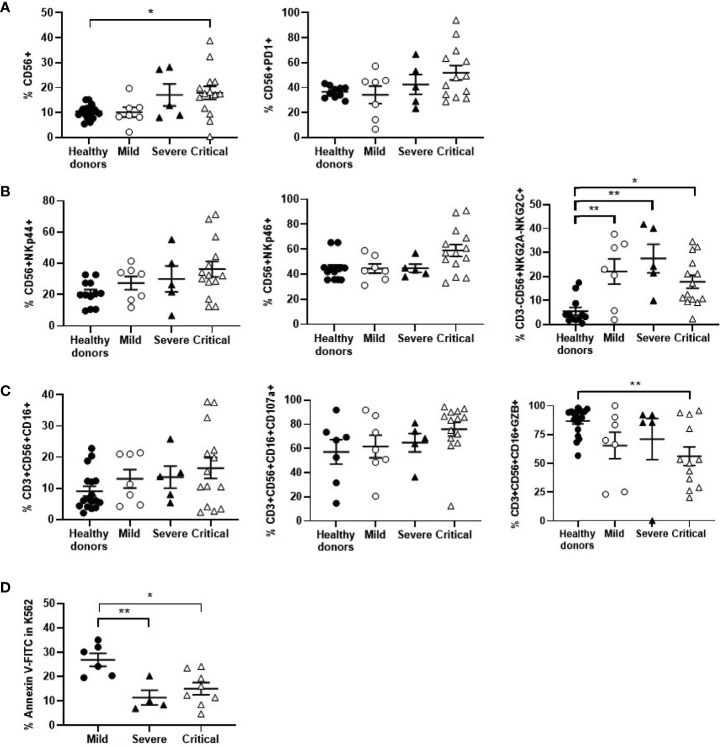
Analysis of NK and NKT cells in patients with different presentations of COVID-19. Analysis of NK cells expressing the activation marker CD56 and the immune exhaustion marker PD1 **(A)**, as well as other activation markers **(B)**. **(C)** Analysis of NKT cell population CD3+CD56+CD16+, as well as the degranulation marker CD107a and their ability to synthesize GZB in response to Hsp70 peptide. **(D)** Cytotoxicity of PBMCs from patients with different presentations of COVID-19 was analyzed by quantifying early apoptosis in K562 cells as unspecific target after co-culture (1:10) for 1 hour. Each dot corresponds to one sample and lines represent mean ± SEM. Statistical significance was calculated using one-way ANOVA and Tukey’s multiple comparisons test. *p < 0.05; **p < 0.01.

The levels of NK cells expressing the activation marker CD56+ were increased 1.8-fold in patients with critical COVID-19 in comparison with mild COVID-19 and healthy donors (p<0.05). However, these cells showed an exhausted phenotype, with the expression of PD1 increased 1.5-fold on the cell surface (**Figure 4C**). Besides, NK cells from critical COVID-19 patients also expressed other activating markers such as NKp44 and NKp46 that were increased 1.3-fold, in comparison with mild patients, whereas NKG2C was overall increased in all patients with COVID-19 (**Figure 4B**). The percentage of NKT cells with cytotoxic phenotype CD3+CD56+CD16+ was increased 1.3-fold in patients with critical COVID-19. The expression of the degranulation marker CD107a was increased 1.2-fold in cells from patients with critical COVID-19, in comparison with mild COVID-19, but the capacity of these cells to synthesize GZB in response to Hsp70 peptide was reduced 1.2-fold (**Figure 4**). Moreover, cytotoxic activity of NK and NKT cells from PBMCs of patients with severe and critical COVID-19 against target cells without MHC class I molecules (missing-self) such as K562 was reduced 2.4- and 1.8-fold, respectively, in comparison with mild COVID-19 (**Figure 4D**).

### HLA-E and KIR Genotyping in Patients With COVID-19

**Figure 5:**
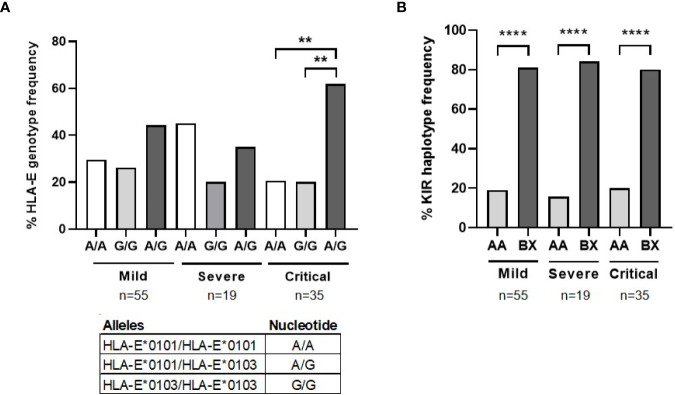
HLA-E and KIR genotyping in patients with different presentations of COVID-19. **(A)** Analysis by qPCR of the percentage of HLA-E genotyping and alleles frequency in patients with severe (n=19) and critical (n=35) COVID-19, in comparison with patients with mild COVID-19 (n=55). **(B)** Analysis of the percentage of KIR alleles and haplotypes frequency in the same patients. Statistical significance was calculated using one-way ANOVA and Tukey’s multiple comparisons test. **p < 0.01; ****p < 0.001.

HLA-E and KIR genotyping was performed due to their importance in the functionality of NK, NKT and TCRγδ+ cells. Similar proportion of homozygosis for HLA-E*0101 (27.9%) and HLA-E*0103 (26.2%) was found in patients with mild COVID-19 (**Figure 5A**), whereas HLA-E*0101 homozygosis (47.4%) was 2.3- fold more frequent than HLA-E*0103 homozygosis (21.5%) in patients with severe COVID-19. Besides, the same percentage of HLA-E*0101 and *0103 homozygosis (19.4%) was found in patients with critical COVID-19, in which HLA-E*0101-0103 heterozygosis was predominant (61.1%) (p<0.01). Therefore, HLA-E heterozygosis was 1.3- and 1.9-fold more present in patients with critical COVID-19 regarding patients with mild and severe COVID-19, respectively. Regarding KIR genotyping, there was a predominance of BX haplotype in all groups of patients (p<0.0001) (**Figure 5B**).

### Application of Random Forest for the Evaluation of the Validity of Predictive Biomarkers

**Figure 6:**
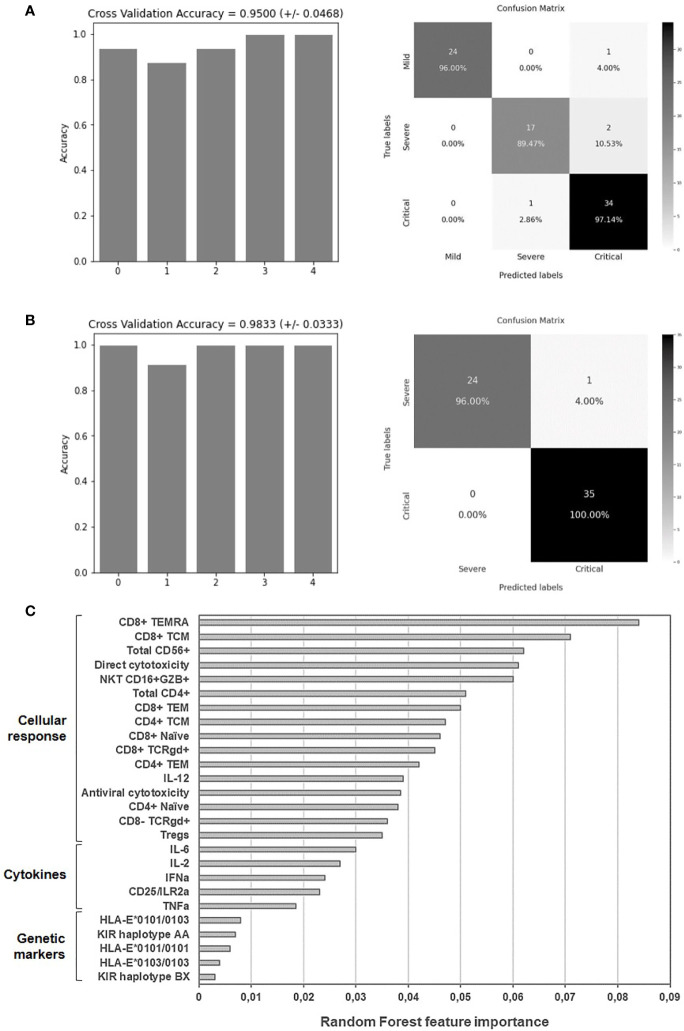
Application of random forest algorithm and Gini VIM method to evaluate the accuracy and importance of the selected biomarkers to predict the worst progression of COVID-19. Accuracy for the 5 iterations of the outer loop of the nested K-fold cross validation and confusion matrix confronting the conditions predicted by the algorithm and the true severity-related conditions of patients with mild, severe and critical COVID-19 **(A)** or with severe and critical COVID-19 **(B)**. **(C)** Relative importance of the selected immunological parameters for the categorization of patients with mild, severe and critical COVID-19, according to Gini VIM method.

The Random Forest algorithm was applied to evaluate the accuracy to predict the categorization of patients with COVID-19 according to those immunological parameters that showed significant differences (p<0.05) between groups. An accuracy of 95.00 ± 4.68% was obtained for the 5 iterations of the outer loop of the nested K-fold cross validation for each competing algorithm (**Figure 6A**). Therefore, 24 out of 25 patients with mild COVID-19 (96.00%), 17 out of 19 patients with severe COVID-19 (89.47%) and 34 out of 35 patients with critical COVID-19 (97.14%) were correctly assigned to the right group using these features. Cross validation accuracy was improved to 98.33±3.33% when only data from severe and critical COVID-19 were analyzed, and then the model predicted patients with critical COVID-19 with 100% of accuracy (35 out of 35 patients) and severe COVID-19 with 96% of accuracy (24 out of 25 patients) (**Figure 6B**). Proportionally, all groups attained similar accuracy in the results even although the number of samples available to perform the training process was different between groups. The Gini VIM (variable importance measure) method determined that variations in immune cells populations such as effector CD8+ T cells, NK and NKT cells, as well as their cytotoxic activity, were more important for the categorization of patients with COVID-19 than the levels of plasma cytokines or the assayed genetic parameters (**Figure 6C**).

## Discussion

The broad variety of clinical presentations of COVID-19 suggests differences in the immune response during the progression of the disease. However, it is unclear why some patients respond so dramatically against the virus whereas in other patients there is a mild or asymptomatic disease. Moreover, the lack of knowledge about the reason for the different presentations of COVID-19 makes really difficult to predict the outcome of the infection and consequently, which are the most useful measures for the clinical management of the patients.

In this study, we recruited 109 patients with different presentations of COVID-19 who were infected during the first peak of transmission in Madrid, one of the major pandemic hotspots in Spain. At that time, Spain was one of the most affected countries in Europe and it was severely hit by the second wave during the second semester of 2020 (32). The death rate caused by COVID-19 in Spain has drawn attention about the possible causes for the severe progression of this infectious disease within the population. In fact, 31% of patients with critical COVID-19 from our cohort deceased during hospitalization. It is still unclear why lethality by SARS-CoV-2 was higher in Spain than in other European countries with similar population density. One of the potential reasons might be that Spain has one of the highest life expectancies among populations worldwide, and therefore the chance to progress to severe disease for patients over 65 years old is high (33). Besides, older patients may not be candidates for intensive measures. Secondly, the population density in Spain is quite high in the major cities, with most people living in crowded small flats instead of individual houses, which would not permit strict isolation. Thirdly, there is a culture of physical closeness among relatives and friends that makes easier the intrafamily spread. And finally, the pandemic has demonstrated the weaknesses of an already deteriorated Healthcare system, as well as poor coordination between authorities (34). In fact, due to the situation of collapse at the time, there was a dramatic lack of ICU beds.

Although high viral load may contribute to a certain degree to the pathogenicity displayed by SARS-CoV-2 (35), several studies revealed a pathogenic relationship between this viral infection and a disproportionate immune response that was responsible for a hyperinflammatory state (36), characterized by high levels of proinflammatory cytokines in the plasma of patients with the most severe forms of COVID-19 (30). Therefore, the characterization of the immune-mediated antiviral mechanisms that are activated during SARS-CoV-2 infection is essential to determine COVID-19 evolution. In this study, we evaluated immunological parameters related to the most critical disease progression so that predictive biomarkers can be defined.

Patients with more severe forms of COVID-19 showed high levels of NK cells with increased expression of activation markers. However, these cells also expressed high levels of the immune exhaustion marker PD1, as well as low cytotoxic activity against target cells, indicating that the immune system was unable to control the progression of the infection despite triggering a potent inflammatory response. This low cytotoxicity reflected an impaired immune response in critical patients, which was supported by low levels of antiviral cytokines in plasma such as IFNα and β. This defective innate immune response has been associated with a poor outcome (37) due to the central role of these cytokines in the antiviral response (38). Corticoids are the only drugs that have proved to be successful for the treatment of the hyperinflammatory state of COVID-19, inducing a significant reduction of mortality, independently of age and gender (39, 40). Accordingly, most hospitalized patients in our cohort were treated with corticoids. Although we cannot completely rule out that treatment with these immunosuppressive drugs may have altered the analysis of the cytotoxic response, we have to consider first, that hospitalized patients with critical COVID-19 still showed higher levels of cytotoxic cells such as CD8 and NK cells than patients with mild disease, and second, that PBMCs from hospitalized patients who were not treated with corticoids also showed an impaired cytotoxic response.

On the other hand, the aberrant release of pro-inflammatory cytokines and chemokines from abortively infected macrophages and dendritic cells may also contribute to the hyperinflammatory state without causing an effective antiviral response (41), mostly due to defective antigen presentation to T cells but also to CD4 lymphopenia. High levels of IL-6, TNFα and IL-8/CXCL8 detected in severe and critical COVID-19 may not only be responsible for the recruitment of neutrophils and cytotoxic T cells (42–44) but also for the immune exhaustion developed during critical COVID-19 (45). The release of chemokines such as CXCL-10/IP-10 and CCL-2/MCP-1 may also contribute to CD4 lymphopenia, mostly due to the suppression of hematopoietic progenitor cells proliferation (46), or to the inhibition of IL-2 signaling pathway (47). In fact, IL-2 levels were reduced in our hospitalized patients, but also Tregs count and sIL-2R/CD25 levels were significantly increased. Finally, effector subsets of CD4+ T cells TEM and TEMRA were in higher proportion in severe and critical COVID-19 than mild disease, which may also contribute to the overall decrease of CD4 count.

All these events that impaired the distribution of T cells subsets may also affect the conversion to Th1 phenotype (41), which is mostly involved in viral clearance through virus-specific adaptive immunity. Shift from Th2 to Th1 response is characterized by increasing levels of IL-1β, IL-2, IL-12, TNFα, and IFNγ in plasma (48), but these cytokines, except for TNFα, were only increased in mild COVID-19, whereas in severe and critical COVID-19 only TNFα was significantly increased, likely contributing to the cytokine storm (49). IL-12 is not only essential for Th1 polarization, but also for CD8+ T cell differentiation (50). Despite the significant reduction in the expression of IL-12 in severe and critical COVID-19, we did not observe CD8 lymphopenia in severe or critical patients, but even a slight increase of CD8+ T cell count in comparison with mild COVID-19. There was also a higher proportion of effector CD8+ TEM and TEMRA in these patients. However, the overall cytotoxic activity against cells infected with pseudotyped SARS-CoV-2 was greatly reduced, probably due to an impaired CD8+ T cell activity. This was in accordance with the low levels of IFN, NK cytotoxic activity and non-conventional T cells expressing TCRγδ observed in these patients. TCRγδ+ T cells, which usually expand in response to cancer or chronic infections such as cytomegalovirus (51), showed a similar cytotoxic activity than NK cells, as well as the ability to recognize cells with missing-self and antigens presented by HLA-E or even without previous antigen processing (52). Moreover, 10% of TCRγδ+ lymphocytes in peripheral blood express KIR on the surface such as NK cells (52). However, the overall low cytotoxicity displayed by cells from severe and critical COVID-19 did not seem related to specific KIR genes because there were no changes between the different COVID-19 presentations, despite KIR BX haplotype was predominant in our cohort of patients. In fact, KIR BX is the most common haplotype in Spanish individuals (53). Besides, patients with critical COVID-19 showed higher predominance of HLA-E heterozygosis, which may be related to a more permissive peptide repertoire presentation that is nevertheless not translated to a better cytotoxic response.

One potential limitation of our study is that the median number of days from clinical onset to sampling was different between the groups of patients due to the confinement decreed by the Spanish Government, which prevented the patients with mild COVID-19 to participate in the study until the confinement ended. However, the adaptive immune response is expected to be fully active within 3 weeks after the infection and SARS-CoV-2-specific CD4+ or CD8+ T cell responses, as well as high levels of neutralizing antibodies, are still detectable after 3–4 months post-infection (54). Due to the vast majority of our patients were recruited within this range of 3 weeks to 4 months post-infection, the immune response data should be comparable between groups. On the other hand, there was an imbalance between the age and gender of patients with mild COVID-19 and those with more severe forms of the disease, due to the most affected individuals in the general population were males over 65 year-old. Therefore, although this imbalance may be considered a bias in the study, our cohorts are more representative of the part of the population that is more severely affected by SARS-CoV-2 infection.

In conclusion, patients hospitalized in Madrid (Spain) due to COVID-19 showed a hyperinflammatory state with low cytotoxic response. Several potential biomarkers of poor prognosis were detected, mostly related to cellular immune response. Changes in the levels of effector CD8+ T cells, NK and NKT cells and their cytotoxic activity showed the highest importance for the prognosis of patients with severe and critical COVID-19 and they correlated with their categorization with 98% of accuracy. Therefore, preventive measures against the progression to a critical form of COVID-19 should not only be directed against the hyperinflammatory state, but also to enhance the cytotoxic response in order to decrease the viral replication and spread.

## Data Availability Statement

The original contributions presented in the study are included in the article/**Supplementary Material**. Further inquiries can be directed to the corresponding authors.

## Ethics Statement

The individuals participating in this study who presented severe and critical forms of COVID-19 were recruited from Hospital Universitario Ramόn y Cajal, Hospital Universitario Puerta de Hierro and Hospital de El Escorial (Comunidad de Madrid, Spain). Individuals with mild COVID-19 were recruited at the Primary Healthcare Center Doctor Pedro Lain Entralgo (Alcorcόn, Madrid, Spain). All individuals gave informed written consent to participate in the study, or witnessed oral consent with written consent by representative to avoid handling of contaminated documents. Current Spanish and European Data Protection Acts ensured confidentiality and anonymity of all participants. Protocol for this study (CEI PI 32_2020-v2) was prepared in accordance with the Helsinki Declaration and previously reviewed and approved by the Ethics Committees of Instituto de Salud Carlos III (IRB IORG0006384) and all participating hospitals.

## The Multidisciplinary Group of Study of COVID-19 (MGS-COVID)

Contributing members of the Multidisciplinary Group of Study of COVID-19 (MGS-COVID): **Alberto Gomez Bonilla**, Centro de Salud Doctor Pedro Laín Entralgo, Alcorcόn, Spain; **Alejandro Luna de Abia**, Hematology Service, Hospital Universitario Ramόn y Cajal, Madrid, Spain; **Ana Corronchano Garcıa**, Internal Medicine Service, Hospital Universitario Severo Ochoa, Leganés, Spain; **Andrea Vinssac Rayado**, Centro de Salud Doctor Pedro Laín Entralgo, Alcorcόn, Spain; **Aurora Expόsito Mora**, Centro de Salud Doctor Pedro Laín Entralgo, Alcorcόn, Spain; **Belén Comeche**, Infectious Diseases Service, Hospital Universitario Ramόn y Cajal, Madrid, Spain; **Claudia Patrias Fernández Fernández**, Internal Medicine Service, Hospital Universitario Severo Ochoa, Leganés, Spain; **Crus Soriano**, Intensive Medicine Service, Hospital Universitario Ramόn y Cajal, Madrid, Spain; **Esther Alonso Herrador**, Centro de Salud Doctor Pedro Laín Entralgo, Alcorcόn, Spain; **Gema Carrillo Blanco**, Internal Medicine Service, Hospital Universitario Severo Ochoa, Leganés, Spain; **Gema Lora Rey**, Centro de Salud Doctor Pedro Laín Entralgo, Alcorcόn, Spain; **Javier Pérez Gonzalez**, Centro de Salud Doctor Pedro Laín Entralgo, Alcorcόn, Spain; **José Antonio Barbado Albadalejo**, Internal Medicine Service, Hospital Universitario Severo Ochoa, Leganés, Spain; **Jose Sanchez Hernández**, Centro de Salud Doctor Pedro Laín Entralgo, Alcorcόn, Spain; Lorena Cordova Castaño, Centro de Salud Doctor Pedro Laín Entralgo, Alcorcόn, Spain; **Lourdes Sampablo Valverde**, Internal Medicine Service, Hospital Universitario Severo Ochoa, Leganés, Spain; **Maria Luisa Muñoz Balsa**, Centro de Salud Doctor Pedro Laín Entralgo, Alcorcόn, Spain; **María Luisa Pinilla Pardo**, Internal Medicine Service, Hospital Universitario Severo Ochoa, Leganés, Spain; **María Mercedes Gea Martinez**, Centro de Salud Doctor Pedro Laín Entralgo, Alcorcόn, Spain; **María Victoria Leon Gomez**, Centro de Salud Doctor Pedro Laín Entralgo, Alcorcόn, Spain; **Pablo Amich Alemany**, Centro de Salud Doctor Pedro Laín Entralgo, Alcorcόn, Spain; **Patricia Mínguez**, Neumology Service, Hospital Universitario Puerta de Hierro, Majadahonda, Spain; **Sandra Arévalo Camacho**, Internal Medicine Service, Hospital Universitario Severo Ochoa, Leganés, Spain; **Sandra Chamorro**, Hematology Service, Hospital Universitario Ramόn y Cajal, Madrid, Spain; **Sandra Pérez-Santos**, Centro de Salud Doctor Pedro Laín Entralgo, Alcorcόn, Spain; **Susana Domínguez-Mateos**, Centro de Salud Doctor Pedro Laín Entralgo, Alcorcόn, Spain; **Susana González Escobar**, Internal Medicine Service, Hospital Universitario Severo Ochoa, Leganés, Spain; **Valle Falcones**, Neumology Service, Hospital Universitario Puerta de Hierro, Majadahonda, Spain; **Victoria Bosch Martos**, Centro de Salud Doctor Pedro Laín Entralgo, Alcorcόn, Spain; **Yolanda Gutierrez Cacenave**, Internal Medicine Service, Hospital Universitario Severo Ochoa, Leganés, Spain.

## Author Contributions

MCoi and ML-H conceptualized the project with the support of VP. MCoi, ML-H, and LV wrote the manuscript. DF performed the analyses of Random Forest. JGP, LV, and MCoi performed the study of cytotoxicity. LV and MT performed the analysis of cell populations by flow cytometry with technical assistance from EM. ML-H and SR-M performed the analysis of cytokines production. LV performed all genetic analyses. EM and MT processed and stored all blood samples. RM, CN, MM-A, MCor, AS-M and VG-G identified, selected, and recruited the patients, and also collected the blood samples, with the invaluable collaboration of all member of MGS-COVID. VG-G, MCor, RM, CN, MM-A and ML-H collected and analyzed the clinical data. All authors contributed to the article and approved the submitted version.

## Funding

This work was supported by the Coordinated Research Activities at the Centro Nacional de Microbiología (CNM, Instituto de Salud Carlos III) (COV20_00679) to promote an integrated response against SARS-CoV-2 in Spain (Spanish Ministry of Science and Innovation) that is coordinated by Dr. Inmaculada Casas (WHO National Influenza Center of the CNM); a generous donation provided by Chiesi España, S.A.U. (Barcelona, Spain); the Spanish Ministry of Economy and Competitiveness (PID2019-110275RB-I00); the Spanish AIDS Research Network RD16CIII/0002/0001 that is included in Acciόn Estratégica en Salud, Plan Nacional de Investigaciόn Científica, Desarrollo e Innovaciόn Tecnolόgica 2016-2020, Instituto de Salud Carlos III, European Region Development Fund (ERDF). The work of ML-H and SR-M is financed by NIH grant R01AI143567. The work of LH is supported by a pre-doctoral grant from Instituto de Salud Carlos III (FIS PI16CIII/00034-ISCIII-FEDER).

## Conflict of Interest

The authors declare that the research was conducted in the absence of any commercial or financial relationships that could be construed as a potential conflict of interest.
