# Supplementary material for: Impaired Cytotoxic Response in PBMCs From Patients With COVID-19 Admitted to the ICU: Biomarkers to Predict Disease Severity
Source: Front Immunol. 2021 May 26;12:665329. doi: 10.3389/fimmu.2021.665329 (PMC8187764; doi:10.3389/fimmu.2021.665329)
Supplement: Supplementary file 1 [file Table_1.docx]

**Supplemental Table 1.** Clinical characteristics of non-hospitalized patients with COVID-19 who were recruited for this study at the Primary Healthcare Center Laín Entralgo (Madrid, Spain).

| **Patient's ID** | **Age (years)** | **Gender** | **Days from clinical onset to sample** | **Exitus** | **Cough and expectoration** | **Dyspnea** | **Fever** | **Pneumonia** | **Bilateral pneumonia** | **Diarrhea and vomiting** | **Lethargy** | **Migraine** | **Asthenia** | **Treatment** | **DM** | **DL** | **HT** |
| --- | --- | --- | --- | --- | --- | --- | --- | --- | --- | --- | --- | --- | --- | --- | --- | --- | --- |
| **1** | 64 | M | 75 | No | Yes | Yes | Und | No | No | Und | No | No | Yes | HCQ | Yes | Yes | No |
| **2** | 57 | W | 85 | No | Yes | Yes | Yes | Yes | Und | Yes | No | No | Yes | HCQ | No | No | No |
| **3** | 58 | W | 73 | No | No | No | No | No | No | No | No | No | Yes | NA | No | No | No |
| **4** | 50 | W | 81 | No | Yes | No | Yes | No | No | No | No | No | Yes | NA | No | No | No |
| **5** | 59 | W | Und | No | No | No | Yes | No | No | No | No | No | Yes | NA | No | Yes | No |
| **6** | 53 | W | 87 | No | Yes | No | Und | No | No | No | No | No | Yes | NA | No | No | No |
| **7** | 41 | W | 83 | No | Yes | No | Yes | No | No | No | No | Yes | Yes | NA | No | No | No |
| **8** | 29 | M | 87 | No | No | No | Yes | No | No | No | No | No | Yes | NA | No | No | No |
| **9** | Und | M | Und | No | No | No | Und | No | No | No | No | No | No | NA | No | No | No |
| **10** | 26 | W | 87 | No | Yes | Yes | Yes | No | No | No | No | No | Yes | LMWH | No | No | No |
| **11** | 26 | M | 88 | No | No | No | No | No | No | Yes | No | No | Yes | NA | No | No | No |
| **12** | 28 | M | 79 | No | Yes | No | Yes | No | No | No | No | No | Yes | NA | No | No | No |
| **13** | 31 | M | 90 | No | No | Yes | Und | Yes | No | Yes | No | No | Yes | NA | No | No | No |
| **14** | 47 | W | 99 | No | Yes | Yes | Yes | No | No | Yes | Yes | Yes | Yes | HCQ, LMWH | No | No | Yes |
| **15** | 62 | M | 82 | No | Yes | Yes | Yes | No | No | No | No | No | Yes | HCQ | No | No | No |
| **16** | 52 | W | 72 | No | Yes | No | Yes | Und | Und | No | Und | No | Yes | NA | No | No | No |
| **17** | 57 | W | 66 | No | No | No | Yes | No | Und | Yes | Und | Yes | Yes | NA | No | Yes | No |
| **18** | 29 | W | 83 | No | Yes | Yes | No | No | No | No | No | No | Yes | NA | No | No | No |
| **19** | 45 | M | 82 | No | Yes | No | Yes | No | No | No | No | Yes | Yes | NA | No | Yes | No |
| **20** | 27 | M | 85 | No | No | No | Yes | No | No | Yes | No | No | Yes | NA | No | No | No |
| **21** | 26 | W | 74 | No | No | No | Yes | Und | Und | No | No | No | No | NA | No | No | No |
| **22** | 25 | W | 61 | No | No | No | No | Und | Und | No | No | No | Yes | NA | No | No | No |
| **23** | 50 | W | 71 | No | Yes | YesÍ | No | Yes | No | Yes | No | Yes | Yes | HCQ | No | No | Yes |
| **24** | 27 | W | 81 | No | Yes | No | Und | Und | Und | No | No | No | No | NA | No | No | No |
| **25** | 32 | M | 95 | No | Yes | No | Yes | Und | Und | Yes | No | Yes | No | NA | No | No | No |
| **26** | 28 | M | Und | No | No | No | No | Und | Und | No | No | No | No | NA | No | No | No |
| **27** | Und | W | 97 | No | No | No | Yes | Und | Und | Yes | No | Yes | Yes | NA | No | No | No |
| **28** | 63 | W | Und | No | No | No | Und | Und | Und | No | No | No | No | NA | No | No | Yes |
| **29** | 48 | W | Und | No | Yes | No | Und | No | Und | No |  | No | No | NA | No | Yes | No |
| **30** | 38 | W | 97 | No | Yes | Yes | Yes | No | No | No | No | Yes | Yes | NA | No | No | No |
| **31** | 25 | W | 99 | No | Yes | No | Yes | Und | Und | Yes | Yes | Yes | Yes | NA | No | Yes | No |
| **32** | 54 | W | Und | No | No | No | Und | No | No | No | No | No | No | NA | No | No | Yes |
| **33** | 59 | M | 107 | No | No | No | Und | No | No | No | No | No | No | NA | No | No | No |
| **34** | 50 | W | Und | No | No | No | Und | No | No | No | No | No | No | NA | No | No | No |
| **35** | 58 | W | 95 | No | No | Yes | Yes | No | No | Yes | No | No | Yes | HCQ | No | Yes | No |
| **36** | 71 | W | 95 | No | Yes | No | Yes | No | No | Yes | No | No | Yes | NA | No | Yes | Yes |
| **37** | 64 | W | 95 | No | Yes | No | Yes | No | No | No | No | No | Yes | NA | No | Yes | No |
| **38** | Und | M | 95 | No | Yes | No | No | No | No | No | No | No | Yes | NA | No | Yes | No |
| **39** | 39 | W | 95 | No | No | Yes | No | Yes | No | No | No | Yes | No | HCQ | No | No | No |
| **40** | 30 | W | 96 | No | No | Yes | Yes | No | No | Yes | No | No | Yes | NA | No | No | No |
| **41** | 26 | M | 91 | No | No | No | Yes | No | No | No | No | Yes | Yes | NA | No | No | No |
| **42** | 30 | W | 88 | No | Yes | Yes | Yes | No | No | Yes | No | Yes | Yes | NA | No | No | No |
| **43** | 59 | W | 90 | No | Yes | Yes | Yes | No | No | No | No | Yes | Yes | NA | No | No | No |
| **44** | 48 | W | 86 | No | No | No | Yes | No | No | No | No | Yes | Yes | NA | No | No | No |
| **45** | 39 | W | Und | No | No | No | Und | No | No | No | No | No | No | NA | No | No | No |
| **46** | 44 | W | 88 | No | Yes | No | Yes | No | No | No | No | Yes | Yes | NA | No | No | No |
| **47** | 48 | W | 94 | No | Yes | Yes | Yes | No | No | Yes | Yes | Yes | Yes | NA | No | No | No |
| **48** | 38 | M | Und | No | No | No | Yes | No | No | No | No | No | No | NA | No | No | No |
| **49** | 47 | M | 97 | No | No | No | Yes | No | No | No | No | Yes | Yes | NA | No | No | No |
| **50** | 54 | W | 84 | No | No | No | Yes | No | No | Yes | No | No | No | NA | No | No | No |
| **51** | 28 | M | 36 | No | Yes | No | Yes | No | No | Yes | No | No | Yes | NA | No | No | No |
| **52** | 48 | W | Und | No | Yes | Yes | Yes | Yes | Yes | No | No | Yes | Yes | NA | No | No | No |
| **53** | 35 | W | 89 | No | Yes | No | No | No | No | Yes | No | Yes | Yes | NA | No | No | No |
| **54** | 36 | W | 95 | No | No | No | No | No | No | Yes | No | Yes | Yes | NA | No | No | Yes |
| **55** | 70 | M | Und | No | No | No | Und | No | No | No | No | No | No | NA | No | No | Yes |

M: man; W: women; Und: undetermined; NA: not applicable; HCQ: hydroxychloroquine; LMWH: Low-molecular-weight heparin; DM: Diabetes mellitus, DL: dyslipidemia; HT: hypertension
