# Supplementary material for: Impaired Cytotoxic Response in PBMCs From Patients With COVID-19 Admitted to the ICU: Biomarkers to Predict Disease Severity
Source: Front Immunol. 2021 May 26;12:665329. doi: 10.3389/fimmu.2021.665329 (PMC8187764; doi:10.3389/fimmu.2021.665329)
Supplement: Supplementary file 2 [file Table_2.docx]

**Supplemental Table 2.** Clinical characteristics of hospitalized patients with severe and critical COVID-19 who were recruited for this study at the Hospitals Ramon y Cajal, Puerta de Hierro and El Escorial (Madrid, Spain).

| **Patient's ID** | **COVID-19** | **Age (years)** | **Gender** | **Days from clinical onset to sample** | **Exitus** | **Cough and expectoration** | **Dyspnea** | **Fever** | **Pneumonia** | **Diarrhea and vomiting** | **Lethargy** | **Migraine** | **Asthenia** | **Treatment** | **DM** | **DL** | **HT** | **DIC** |
| --- | --- | --- | --- | --- | --- | --- | --- | --- | --- | --- | --- | --- | --- | --- | --- | --- | --- | --- |
| **56** | Severe | 66 | M | Und | No | Und | Und | Yes | Yes | Und | Und | Und | Und | HCQ, LPV/r, AZM, CS | No | No | No | No |
| **57** | Severe | 77 | M | 35 | No | No | Yes | Yes | Yes | No | No | No | Yes | HCQ, LPV/r, AZM, TOZ, CS | Yes | Yes | Yes | No |
| **58** | Severe | 63 | W | 14 | No | Und | Yes | No | No | Yes | No | No | Yes | HCQ, LPV/r, AZM, CS | No | No | Yes | No |
| **59** | Severe | 72 | M | 28 | No | Yes | No | Yes | No | No | No | No | Yes | HCQ, AZM, CS | No | No | Yes | No |
| **60** | Severe | 72 | M | 40 | No | Yes | No | Yes | Yes | No | No | No | No | HCQ LPV/r, TOZ, CS | Yes | Yes | Yes | Yes |
| **61** | Severe | 72 | M | 10 | No | No | No | Yes | Yes | No | No | No | No | HCQ, LPV/r, AZM, LMWH, TOZ, CS | No | Yes | No | Yes |
| **62** | Severe | 67 | W | 18 | No | No | No | Yes | Yes | No | No | No | Yes | HCQ, LPV/r, AZM, LMWH, TOZ, CS | No | No | No | No |
| **63** | Severe | 80 | M | Und | No | Und | Yes | Und | Yes | Und | Yes | Und | Und | HCQ, AZM, CS | Yes | No | Yes | Yes |
| **64** | Severe | 59 | W | 32 |  | Yes | Yes | Yes | No | No | No | No | No | HCQ, AZM | Yes | Yes | No | No |
| **65** | Severe | 72 | W | 1 | No | Yes | Yes | No | Yes | Yes | No | No | No | HCQ, AZM, TOZ, CS | No | No | No | No |
| **66** | Severe | 73 | M | 42 | No | Yes | No | No | Yes | No | No | No | No | LPV/r, AZM, TOZ, CS | No | No | Yes | No |
| **67** | Severe | 84 | W | 52 | No | Yes | No | No | Yes | No | No | Yes | No | HCQ, LPV/r, AZM, CS | No | No | Yes | No |
| **68** | Severe | 99 | W | 37 | No | Yes | No | No | Yes | No | No | No | No | HCQ, AZM, LMWH | Yes | No | Yes | No |
| **69** | Severe | 84 | W | Und | No | No | No | No | Yes | No | No | No | No | HCQ, AZM |  | Yes | Yes | No |
| **70** | Severe | 69 | M | 17 | No | No | No | Yes | Yes | No | No | No | No | HCQ, TOZ, CS | No | No | Yes | S |
| **71** | Severe | 89 | M | Und | No | No | No | No | Yes | Yes | No | No | No | HCQ | No | No | Yes | Yes |
| **72** | Severe | 50 | M | 3 | No | Yes | No | No | Yes | No | No | No | No | HCQ | No | No | Yes | No |
| **73** | Severe | 90 | M | Und | No | 90 | Yes | No | No | No | No | No | No | HCQ, AZM, LMWH, CS | No | No | No | No |
| **74** | Severe | 76 | M | 25 |  | Yes | No | No | Yes | No | No | No | No | HCQ, LPV/r, AZM, LMWH | No | Yes | Yes | No |
| **75** | Critical | 58 | M | 43 | No | Yes | Yes | Und | Yes | No | No | No | Yes | HCQ, LPV/r, AZM, TOZ, CS | No | Yes | Yes | Yes |
| **76** | Critical | 77 | M | 45 | No | Yes | No | Yes | Yes | No | Yes | No | No | HCQ, LPV/r, AZM, IFN, TOZ, CS | Yes | Yes | Yes | Yes |
| **77** | Critical | 52 | M | 50 | No | No | Yes | Yes | Yes |  | No | Yes | No | HCQ, LPV/r, /AZM, TOZ, CS | No | No | Yes | No |
| **78** | Critical | 66 | W | Und | No | No | No | Yes | Yes | No | Yes | No | No | Und | No | Yes | Yes | No |
| **79** | Critical | 59 | M | 44 | No | Yes | Yes | Yes | Yes | No | No | Yes | No | HCQ, LPV/r, AZM, LMWH, TOZ, CS | No | No | Yes | No |
| **80** | Critical | 50 | M | 48 | No | Yes | No | No | Yes | No | Yes | Yes | No | HCQ, LPV/r, AZM, LMWH, CS | No | No | Yes | No |
| **81** | Critical | 56 | M | 39 | No | Yes | Yes | Yes | Yes | No | No | Yes | No | HCQ, LPV/r, AZM, LMWH, TOZ, CS | No | No | Yes | No |
| **82** | Critical | 65 | M | 62 | No | Yes | Yes | Yes | Yes | No | No | Yes | No | HCQ, LPV/r, AZM, LMWH, TOZ, CS | No | Yes | Yes | No |
| **83** | Critical | 72 | W | 55 | No | No | Yes | Yes | No | No | No | No | No | HCQ, LPV/r, AZM, LMWH, CS | No | No | Yes | No |
| **84** | Critical | 58 | W | 38 | No | Yes | Yes | Yes | Yes | Yes | No | Yes | Yes | HCQ, LPV/r, AZM, LMWH, TOZ, CS | No | No | No | No |
| **85** | Critical | 56 | W | 18 | No | Yes | No | No | Yes | No | No | No | No | HCQ, LPV/r, LMWH, CS | No | No | No | No |
| **86** | Critical | 69 | M | 55 | No | Yes | Yes | Yes | Yes | No | No | Yes | No | HCQ, LPV/r, AZM, LMWH, TOZ, CS | No | No | No | No |
| **87** | Critical | 62 | M | 50 | Yes | Yes | Yes | Yes | Yes | No | No | Yes | No | HCQ, LPV/r, AZM, LMWH, TOZ, CS | No | Yes | No | No |
| **88** | Critical | 63 | W | 46 | Yes | No | No | No | No | No | No | No | No | HCQ, LMWH, CS | No | No | No | No |
| **89** | Critical | 52 | M | 34 | Yes | Yes | Yes | Yes | Yes | No | No | Yes | No | LPV/r, AZM, LMWH, TOZ, CS | No | No | Yes | No |
| **90** | Critical | 57 | M | 53 | Yes | Yes | Yes | Yes | Yes | No | No | No | No | HCQ, LPV/r, AZM, LMWH, TOZ, CS | No | No | Yes | No |
| **91** | Critical | 71 | M | Und | No | Und | Und | Und | Und | Und | Und | Und | Und | LMWH and Und | No | No | No | Und |
| **92** | Critical | 50 | M | 14 | No | Yes | Yes | Yes | Yes | No | No | Yes | No | HCQ, LPV/r, AZM, LMWH, CS | No | No | No | No |
| **93** | Critical | 59 | M | Und | No | Und | Und | Und | Und | Und | Und | Und | Und | HCQ, LPV/r, AZM, LMWH, TOZ, CS | No | No | SI | Und |
| **94** | Critical | 72 | W | 59 | No | Yes | Yes | Yes | Yes | No | No | Yes | No | HCQ, LPV/r, AZM, LMWH, TOZ, CS | No | Yes | Yes | No |
| **95** | Critical | 73 | M | 19 | No | Yes | Yes | Yes | Yes | No | No | No | No | HCQ, LPV/r, AZM, LMWH, TOZ, CS | No | Yes | No | No |
| **96** | Critical | 73 | M | Und | No | No | No | No | Yes | No | No | Yes | No | HCQ, AZM, LMWH, CS | Yes | Yes | Yes | No |
| **97** | Critical | 65 | M | 25 | No | Yes | Yes | Yes | Yes | No | No | Yes | No | HCQ, LPV/r, AZM, LMWH, TOZ, CS | No | No | No | No |
| **98** | Critical | 62 | M | Und | No | Yes | No | Und | Und | Und | Und | Und | Und | HCQ, LPV/r, AZM, LMWH, TOZ, CS | Yes | No | No | Und |
| **99** | Critical | 73 | W | Und | No | No | No | No | Yes | No | No | No | No | HCQ, LPV/r, AZM, LMWH, TOZ, CS | No | No | Yes | No |
| **100** | Critical | 40 | M | 71 | No | Und | Und | Yes | Yes | Yes | No | No | No | HCQ, AZM, CS | Und | Und | Und | No |
| **101** | Critical | 67 | M | 23 | Yes | No | Yes | Yes | Yes | No | No | No | No | HCQ, AZM, TOZ, CS | No | Yes | Yes | No |
| **102** | Critical | 63 | W | 28 | Und | Yes | Yes | Yes | Yes | No | No | No | No | HCQ, TOZ, CS | No | Yes | No | Yes |
| **103** | Critical | 69 | M | 1 | Yes | Und | Und | Yes | Und | Und | Und | Und | Und | Und | Und | Und | Und | No |
| **104** | Critical | 81 | W | Und | Yes | No | No | No | Yes | No | No | No | No | HCQ, LPV/r, AZM, CS | Yes | Yes | Yes | No |
| **105** | Critical | 64 | M | 53 | Yes | Yes | Yes | Yes | Yes | No | No | No | No | HCQ, LPV/r, AZM, LMWH, TOZ, CS | Yes | Yes | No | No |
| **106** | Critical | 65 | M | No | Yes | Yes | No | No | Yes | No | No | Yes | No | HCQ, LPV/r, AZM, LMWH, CS | No | No | No | No |
| **107** | Critical | 56 | M | 30 | Yes | No | Yes | Yes | No | No | No | No | Yes | HCQ, LMWH, TOZ, CS | No | No | Yes | Yes |
| **108** | Critical | 92 | M | Und | Yes | No | No | No | Yes | No | No | No | Yes | HCQ, AZM | No | No | Yes | No |
| **109** | Critical | 42 | M | 39 | Und | Yes | Yes | Yes | Yes | No | No | No | Yes | HCQ, LPV/r, AZM, LMWH, TOZ, CS | No | No | Yes | Und |

M: man; W: women; Und: undetermined; NA: not applicable; HCQ: hydroxychloroquine; LPV/r: lopinavir/ritonavir; AZM: azithromycin; LMWH: Low-molecular-weight heparin; TOZ: tocilizumab; CS: corticosteroids; DM: Diabetes mellitus, DL: dyslipidemia; HT: hypertension; DIC: Disseminated Intravascular Coagulation

**Supplemental Table 2 (continuation).** Data collected during hospitalization of patients with severe and critical COVID-19 who were recruited for this study at the Hospitals Ramon y Cajal, Puerta de Hierro and El Escorial (Madrid, Spain).

| **Patient's ID** | **COVID-19** | **Hospitalization** | | | | | | | **Blood biochemistry data** | | | | | | | | | | |
| --- | --- | --- | --- | --- | --- | --- | --- | --- | --- | --- | --- | --- | --- | --- | --- | --- | --- | --- | --- |
|  |  | **Days of Hospitalization** | **Days of Hospitalization at sampling** | **Days in ICU** | **NIV** | **IV** | **Reservoir** | **Nasal Glasses** | **CRP (mg/mL)** | **LDH (U/L)** | **CK (U/L)** | **Ferritin (ng/mL)** | **IL-6 (pg/mL)** | **D-dimer (ug/mL)** | **Procalcitonin (ng/mL)** | **Lymphocytes (cels/uL)** | **Monocytes (cels/uL)** | **Platelets (cels/uL)** | **Fibrinogen (mg/dL)** |
| **56** | Severe | 23 | 23 | NA | No | No | No | Yes | 118.8 | 478 | 3337 | 1045 | 8.7 | 2.0 | Und | 300 | 200 | 80000 | 516 |
| **57** | Severe | 67 | 31 | NA | Yes | No | Yes | Yes | 125.1 | 732 | 33 | 1334 | 767.9 | 33.9 | 0.08 | 400 | 200 | 82000 | 166 |
| **58** | Severe | 8 | 2 | NA | No | No | No | No | 104.9 | 3.04 | 61 | 222 | Und | 3.1 | Und | 2850 | 700 | 262000 | 358 |
| **59** | Severe | 24 | 22 | NA | No | No | No | No | 146.3 | Und | 51 | 4821 | Und | 0.6 | 0.15 | 500 | 500 | 116000 | 819 |
| **60** | Severe | 50 | 35 | NA | Yes | No | Yes | Yes | 363.5 | 632 | 102/ 11 | 1033 | 4624 | 10.1 | 1 | 800 | 100 | 190000 | 778 |
| **61** | Severe | 40 | 35 | NA | No | No | Yes | No | 234.8 | 1454 | 93 | 2462 | 1497.8 | 2.7 | 0.05 | 25.8 | 0.3 | 117000 | 785 |
| **62** | Severe | 29 | 17 | NA | Yes | No | No | Yes | 155.5 | 707 | 13 | 622 | 3718.3 | 4.8 | 0.2 | 1000 | 200 | 128000 | 710 |
| **63** | Severe | 38 | 20 | NA | No | No | No | Yes | 156.6 | 449 | 18 | 2374 | Und | 9.9 | Und | 700 | 3200 | 97000 | 694000 |
| **64** | Severe | 10 | 7 | NA | Yes | No | No | Yes | 157 | 581.8 | 37 | 29 | Und | 0.4 | 10 | 4100 | 15000 | 47000 | 570 |
| **65** | Severe | 9 | 25 | NA | Yes | No | No | Yes | 78.2 | 596 | 192 | 620 | 12.3 | 1.0 | 0.37 | 300 | 1100 | 480000 | 573 |
| **66** | Severe | 38 | 32 | NA | No | No | No | Yes | 173 | 489 | 273 | 0.6 | 5855.66 | 3.1 | 0.6 | 390 | Und | 70000 | 195 |
| **67** | Severe | 44 | 42 | NA | Yes | Yes | Yes | Yes | 152 | 878 | 338 | 0.31 | Und | 6.8 | 0.31 | 190 | Und | 106000 | 447 |
| **68** | Severe | 8 | 7 | NA | No | No | No | Yes | 14.6 | 878 | 34 | 0.1 | Und | 1.1 | 0.1 | 1350 | Und | 182000 | 436 |
| **69** | Severe | 7 | 8 | NA | No | No | No | Yes | 10.64 | 465 | 15 | 563 | Und | 5.6 | Und | 820 | 380 | 7400 | Und |
| **70** | Severe | 16 | 11 | NA | Yes | No | No | Yes | 23.4 | 495 | 13 | 2356 | Und | 0.5 | 2.64 | 460 | 1180 | 79000 | Und |
| **71** | Severe | 32 | 15 | NA | No | No | No | No | 29.19 | 463 | 7012 | 1719 | Und | 8.6 | Und | 460 | 1590 | 79000 | Und |
| **72** | Severe | 10 | 5 | NA | No | No | No | No | 113 | 217 | 134 | 0.03 | Und | 1.1 | 0.03 | 190 | 10 | 214000 | 418 |
| **73** | Severe | 13 | 12 | NA | No | No | No | No | 13.52 | 202 | 7 | 1557 | Und | 1.4 | 0.11 | 380 | 40 | 119000 | Und |
| **74** | Severe | 21 | 23 | NA | No | No | No | Yes | 178.4 | 364 | 248 | 1544 | 144.1 | 2.0 | Und | 400 | 1200 | 144000 | 998 |
| **75** | Critical | 45 | 39 | 13 | Yes | Yes | No | Yes | 187.8 | 627 | 2043 | 1101 | 233.6 | 13.1 | Und | 300 | 600 | 247000 | 174 |
| **76** | Critical | 44 | 40 | 10 | Yes | Yes | No | No | 102.3 | 671 | 770 | 582 | Und | 49.9 | 0.07 | 500 | 300 | 669000/ 145000 | 641 |
| **77** | Critical | 73 | 42 | 39 | No | Yes | No | Yes | 115.4 | 553 | 1785 | 1666 | 8.3 | 11.7 | 0.44 | 400 | 100 - 20000 | 76000 | 764 |
| **78** | Critical | 13 | 30 | 10 | Yes | No | No | Yes | 209.5 | 668 | 13-258 | 999 | 74.2 | 5.5 | 0.06 | 600 | 0 | 652-11 | 799 |
| **79** | Critical | 45 | 43 | 7 | No | Yes | Yes | Yes | 360 | 561 | 72 | 9.34 |  | 4.0 | 9.34 | 340 | 40 | 166000 | 309 |
| **80** | Critical | 52 | 44 | 13 | No | Yes | Yes | Yes | 326 | 1096 | 535 | 0.55 | 41.4 | 6.7 | 0.55 | 440 | 260 | 150000 | 519 |
| **81** | Critical | 37 | 35 | 30 | No | Yes | Yes | Yes | 36.8 | 590 | 309 | 0.55 | 134.96 | 5.3 | 0.55 | 210 | 250 | 102000 | 422 |
| **82** | Critical | 110 | 48 | 60 | No | Yes | Yes | Yes | 441 | 1031 | 608 | 13.12 | 353.8 | 6.3 | 13.12 | 110 | 340 | 113000 | 123 |
| **83** | Critical | 94 | 53 | 61 | No | Yes | Yes | Yes | 273.6 | 880 | 347 | 4.34 | 39.85 | 3.2 | 4.34 | 210 | 140 | 12000 | 407 |
| **84** | Critical | 96 | 31 | 42 | No | Yes | Yes | Yes | 276 | 971 | 3517 | 0.72 | 524.8 | 35.0 | 0.72 | 250 | 250 | 55600 | 130 |
| **85** | Critical | 81 | 45 | 37 | No | Yes | Yes | Yes | 231 | 635 | 85 | 2.31 | 24.88 | 35.0 | 2.31 | 410 | 0 | 138000 | 400 |
| **86** | Critical | 110 | 48 | 76 | No | Yes | Yes | Yes | 304 | 548 | 236 | 0.66 | 163.09 | 8.9 | 0.66 | 240 | 230 | 87000 | 414 |
| **87** | Critical | 103 | 48 | 94 | No | Yes | Yes | Yes | 491 | 726 | 236 | 1.78 | 696.49 | 9.5 | 1.78 | 130 | 110 | 48300 | 100 |
| **88** | Critical | 26 | 15 | 54 | No | No | No | Yes | 374 | 900 | 528 | 3.41 | 273.79 | 11.5 | 3.41 | 170 | 50 | 157000 | 277 |
| **89** | Critical | 48 | 28 | 49 | No | Yes | Yes | No | 446 | 987 | 3228 | 22.23 | 2688 | 2.7 | 22.23 | 220 | 280 | 30000 | 288 |
| **90** | Critical | 58 | 51 | 48 | No | Yes | Yes | Yes | 110 | 871 | 433 | 0.3 | 289 | 7.9 | 0.3 | 320 | 200 | 150000 | 152 |
| **91** | Critical | 114 | 72 | 91 | No | Yes | Yes | Yes | Und | Und | Und | Und | Und | Und | Und | Und | NA | Und | Und |
| **92** | Critical | 23 | 8 | 13 | No | Yes | Yes | Yes | 339 | 576 | 238 | 1.68 | 385 | 5.1 | 1.68 | 350 | 140 | 223000 | 340 |
| **93** | Critical | 0 | Und | 63 | No | Yes | Yes | Yes | Und | Und | Und | Und | Und | Und | Und | Und | Und | Und | Und |
| **94** | Critical | 92 | 57 | 66 | No | Yes | Yes | Yes | 311 | 629 | 374 | 11.73 | 1240 | 6.0 | 11.73 | 320 | 240 | 177000 | 217 |
| **95** | Critical | 36 | 13 | 49 | Yes | Yes | Yes | Yes | 104 | 774 | 192 | 0.39 | 476 | 6.1 | 0.39 | 490 | 360 | 80000 | 112 |
| **96** | Critical | Und | 3 | 91 | No | Yes | Yes | Yes | 291 | 286 | 83 | 14.83 | Und | 3.6 | 14.83 | 450 | 100 | 164000 | 437 |
| **97** | Critical | 105 | 51 | 90 | No | Yes | Yes | Yes | 309 | 785 | 869 | 0.35 | 21 | 11.7 | 0.35 | 550 | 150 | 72000 | 243 |
| **98** | Critical | 93 | Und | NA | Und | Und | Und | Und | Und | Und | Und | Und | Und | Und | Und | Und | Und | Und | Und |
| **99** | Critical | 45 | 39 | 16 | No | Yes | Yes | Yes | 317 | 584 | 112 | 0.21 | 1057 | Und | 0.21 | 490 | 140 | 142000 | 445 |
| **100** | Critical | 63 | 60 | 45 | Yes | Yes | No | Yes | 250 | 540 | 274 | 3898 | Und | 8.8 | 1.45 | 300 | 2400 - 200 | 626000 | 1007 |
| **101** | Critical | 29 | 19 | NA | Yes | No | No | No | 115.6 | 1230 | 240 | 4535 | 2232 | 3.0 | 0.33 | 200 | 300 | 151000 | 814 |
| **102** | Critical | 34 | 21 | NA | No | No | No | No | 250 | 801 | 561 | 2688 | Und | 8.6 | Und | 800 | 1200 | 130 | 915 |
| **103** | Critical | 38 | 19 | Und | Und | Und | Und | Yes | 88.9 | 1068 | 139 | 2166 | 960.1 | 3.4 | Und | 400 | 100 | 71000 | 590 |
| **104** | Critical | 27 | 12 | NA | Yes | No | Yes | Yes | 35.9 | 906 | 820 | 799 | Und | 20.0 | 2.54 | 190 | 2090 | 46000 | Und |
| **105** | Critical | 54 | 52 | 56 | Yes | Yes | Yes | Yes | 188 | 120 | 197 | 0.39 | 499 | 8.8 | 0.39 | 380 | 260 | 186000 | 120 |
| **106** | Critical | 60 | 49 | 51 | No | Yes | Yes | Yes | 330 | 817 | 191 | 3.36 | 38.29 | 5.5 | 3.36 | 130 | 170 | 141000 | 422 |
| **107** | Critical | 55 | 41 | 25 | No | Yes | Yes | Yes | 289 | 708 |  | 2.58 | 635 | 5.4 | 2.58 | 0 | 0 | 7000 | 202 |
| **108** | Critical | 17 | 13 | NA | No | No | No | No | 24.85 | 0.28 | 119 | 1101 | Und | 0.6 | Und | 8330 | 280 | 119000 | Und |
| **109** | Critical | 34 | 22 | 26 | Yes | Yes | No | Yes | 112.9 | 374 | 384 | 563 | Und | 8.9 | 0.29 | 200 | 200 | 57000 - 705000 | 703 |

ICU: Intensive Care Unit; NIV: Non-invasive mechanical ventilation; IV: Invasive mechanical ventilation; CRP: C Reactive Protein; LDH: lactate dehydrogenase; CK: Creatine kinase; IL-6: Interleukin-6; Und: Undetermined.
